# Genetic Predisposition in Müllerian Malformations: A Case Report

**DOI:** 10.7759/cureus.86228

**Published:** 2025-06-17

**Authors:** Alejandro Rendón-Molina, Andrea Olguín-Ortega

**Affiliations:** 1 Department of Gynecology, National Institute of Perinatology, Mexico City, MEX

**Keywords:** bicornuate uterus, genetic predisposition, longitudinal vaginal septum, müllerian malformations, pathology regarding infertility

## Abstract

Müllerian malformations (MM) are congenital anomalies of the female reproductive tract that may have a hereditary component. We report the case of a 20-year-old woman with a notable family history of MM (mother and grandmother with a longitudinal vaginal septum and aunt with a bicornuate uterus) who presented with difficulty inserting tampons and menstrual cups. Physical examination revealed a 3 cm longitudinal vaginal septum, which was confirmed by magnetic resonance imaging (MRI) as a 4 cm septum in the lower two-thirds of the vagina, with no abnormalities in the uterus or cervix. Surgical resection was performed without complications, and the patient recovered uneventfully with standard postoperative care. This case points out the relevance of recognizing familial patterns in MM and suggests that early evaluation and counseling may improve reproductive outcomes and minimize associated complications.

## Introduction

Müllerian malformations (MM) are complex and heterogeneous developmental disorders of the embryonic Müllerian duct, which forms most of the female reproductive tract, characterized by diverse clinical presentations without a clear genotype-phenotype correlation [1]. Around six weeks post-fertilization, the terminal ends of both Müllerian ducts fuse to form the uterovaginal duct, which develops into the fornix of the vagina, and by 10 weeks, the fusion of their basement membranes leads to the formation of the uterus, initially divided by a septum that marks the fusion site and is usually resorbed through apoptosis later in development [2].

Genes such as RBM8A and TBX6 have been identified as associated with malformations of the reproductive system; mutations in TBX6 result in mesodermal defects, contributing to uterine malformations such as bicornuate and septate uteri [3]. Alterations in anti-Müllerian hormone receptor (AMHR) genes and variations in the WNT4 gene have been linked to reproductive anomalies, including Mayer-Rokitansky-Küster-Hauser (MRKH) syndrome, resulting in the improper formation of the fallopian tubes and ovarian abnormalities [4].

MM are congenital malformations that occur more frequently than commonly perceived, with a prevalence ranging from 0.5% to 6.7% in the general population and up to 16.7% among women experiencing recurrent miscarriage [5].

Genetic studies aimed at identifying these anomalies may serve as a screening tool for patients with a family history of MM, thereby enabling early diagnosis and reducing the risk of long-term complications, including infertility, pelvic inflammatory disease, and endometriosis [6]. In a final analysis of 1,397 cases, kinship analysis identified 27 family clusters with a mean familial standardized incidence ratio of 3.43 (p < 0.01), indicating that approximately 10% of Müllerian anomalies may be attributable to familial association, with relative risks of 11.6 for first-degree relatives, 8.78 for parents/children, 12.98 for siblings, 1.44 for first cousins, and 1.30 for second cousins [7].

Specific MM

Septate Uterus

Compared with healthy controls, individuals with MM exhibited three hypomethylated CpG sites and increased PAX2 mRNA levels, suggesting that aberrant PAX2 promoter methylation may contribute to Müllerian duct anomaly (MDA) development, while differential methylation in HOXA10, EMX2, TP63, ITGB3, LHX1, GSC, WNT4, and H19 was not associated with changes in their gene expression or disease development [8].

MRKH Syndrome

Recently, genomic studies utilizing chromosomal microarray and genome-wide sequencing have identified several promising genetic variants linked to MRKH syndrome, such as deletions at 17q12 (LHX1, HNF1B) and 16p11.2 (TBX6), as well as sequence variations in GREB1L and PAX8 [9]. The identification of a 16p11.2 microdeletion in 5% of isolated and 8% of syndromic cases highlights its potential role in the pathogenesis of Müllerian aplasia, while the overall high frequency of recurrent copy number variants across all forms underscores important implications for understanding the condition’s etiology and providing genetic counseling to affected families [10]. In another study, the most frequently reported chromosomal regions and potential implicated genes include 1q21.1 (RBM8A), 1p31-1p35 (WNT4), 7p15.3 (HOXA), 16p11 (TBX6), 17q12 (LHX1 and HNF1B), 22q11.21, and Xp22, stating that while the etiology of MRKH syndrome is complex, associated clinical features can help identify specific genetic defects [11].

Bicornuate Uterus

Previously, a polymalformative syndrome presenting with left arm agenesis, a bicornuate uterus, and a bicuspid aortic valve has been reported, in which whole exome sequencing revealed point mutations in HOXA9 and HOXA13, associated with this syndromic phenotype, thereby broadening the known phenotypic spectrum of HOXA-related disorders [12].

Didelphic and Unicornuate Uterus

Coexistence of urogenital abnormalities, such as left kidney agenesis and uterus didelphys, has been reported in association with 15q24 microdeletion syndrome, which is linked to midline defects due to abnormal development [13].

## Case presentation

A 20-year-old female patient presented with significant family history, including a mother and maternal grandmother diagnosed with a longitudinal vaginal septum and a maternal aunt diagnosed with a bicornuate uterus. Her non-pathological personal history includes a healthy diet and high-performance physical activity practiced five times per week. No relevant pathological personal history was noted. In the gynecological and obstetric domain, the patient experienced menarche at the age of 12, with regular menstrual cycles lasting 28 days and a four-day flow, accompanied by mild dysmenorrhea that did not require analgesic management. The patient reported not being sexually active.

Reason for consultation

Three months prior to her consultation, the patient reported difficulty with the placement of menstrual hygiene products, initially tampons and later a menstrual cup, prompting her to seek gynecological evaluation.

Physical examination

During the initial gynecological examination, conducted with the patient’s informed consent, the vulva appeared normal upon inspection. Observation of the introitus revealed a longitudinal vaginal septum approximately 3 cm in length, extending cranially along the vaginal canal. The vaginal length was measured at 9 cm, with no other visible abnormalities during the examination.

Imaging studies

**Figure 1:**
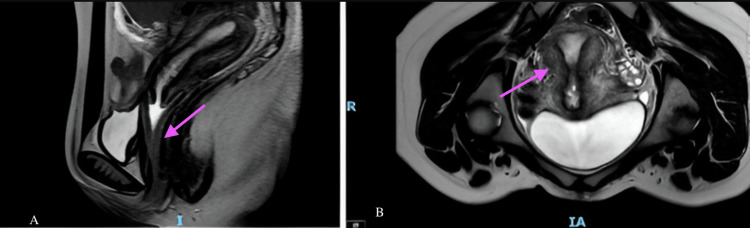
Pelvic MRI Pelvic magnetic resonance imaging (MRI) showing a longitudinal vaginal septum. (A) Sagittal T2-weighted image demonstrating a linear hypointense band (arrow) partially dividing the vaginal canal in the anteroposterior direction. (B) Axial T2-weighted image identifying the septum as a midline hypointense structure (arrow) separating the vaginal lumen into two hemichannels. No abnormalities were observed in the uterus or cervix

A pelvic magnetic resonance imaging (MRI) was performed, revealing a longitudinal vaginal septum extending exclusively through the lower two-thirds of the vagina, with an approximate length of 4 cm. No apparent abnormalities were observed in the cervix or uterus, both of which appeared structurally normal and without duplications (Figure 1), corresponding to a U0C0V1 classification according to the ESHRE classification of female genital tract anomalies [14].

Surgical intervention

**Figure 2:**
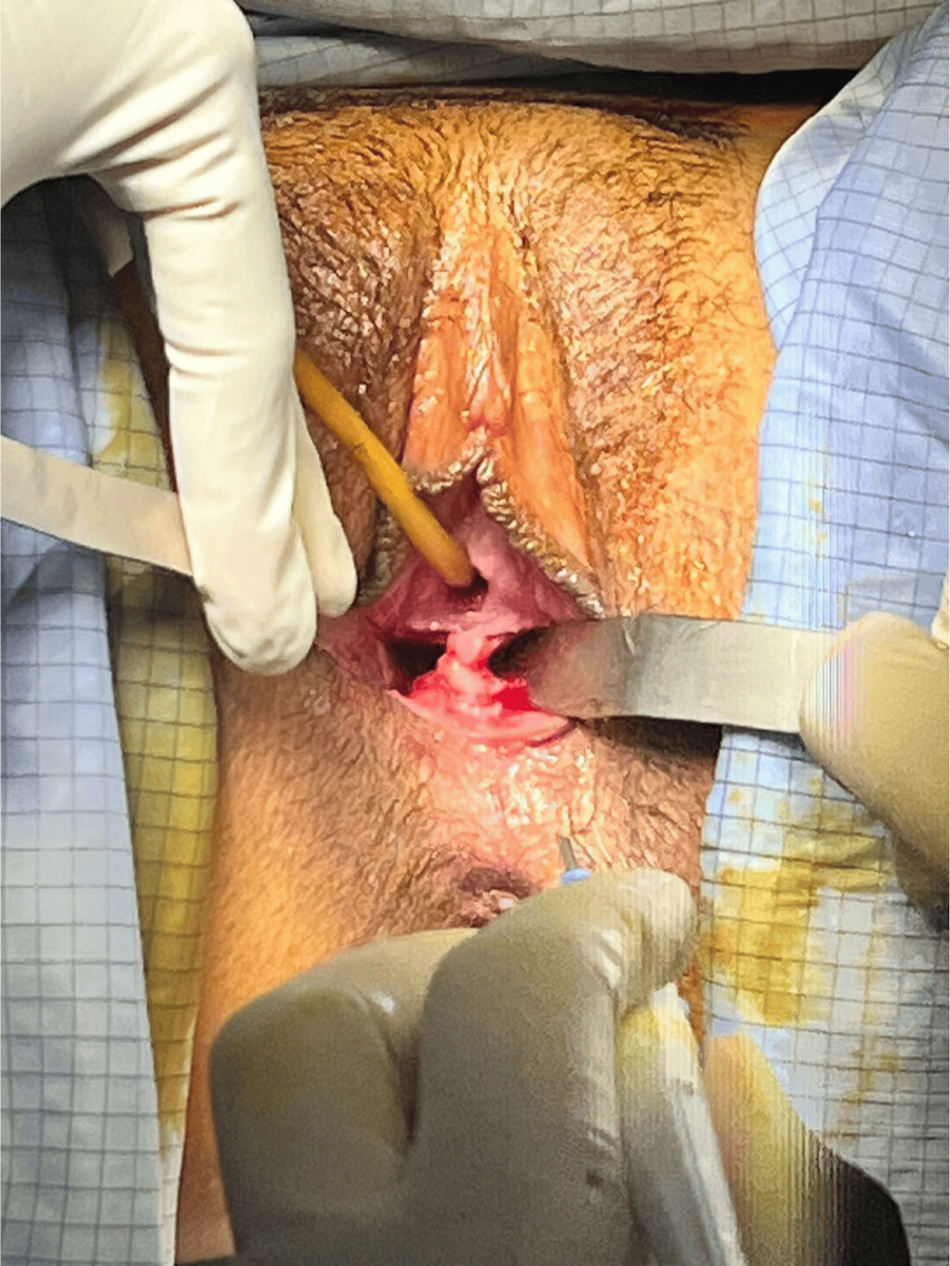
Surgical resection of the vaginal longitudinal septum Intraoperative view showing the central vaginal septum; surgery was performed without complications or technical difficulties

The patient was scheduled for resection of the longitudinal vaginal septum after providing informed consent. During the surgical procedure, the central septum, measuring approximately 4 cm, was successfully resected without complications or technical difficulties (Figure 2). A thorough examination of the vaginal canal confirmed a vaginal length of 9 cm. The cervix was visualized, with no abnormalities or duplications identified. A hysterometry was performed, reporting a single uterine cavity measuring 7 cm in length, confirming the anatomical and functional integrity of the uterus.

Postoperative progress

The patient was discharged on the same day of the procedure with instructions to use a vaginal stent for seven days to prevent scar formation and promote optimal healing. During the postoperative follow-up, conducted one week after the procedure, adequate healing was observed, with symmetrical hymeneal caruncles and no evidence of vaginal stenosis or adhesions.

Follow-up

In the most recent follow-up consultation, the possibility of a genetic component underlying the structural anomalies observed in her family was discussed. A genetic evaluation to identify potential mutations or hereditary patterns was suggested. However, the patient expressed that she does not wish to undergo additional genetic studies at this time but remains informed about the importance of future evaluation should her clinical condition or decision change.

## Discussion

A broad range of anomalies can arise depending on the stage at which Müllerian development halts in utero, varying from mild cases like a partial uterine septum to severe cases such as the complete absence of the cervix, uterus, and fallopian tubes, as seen in Müllerian agenesis [15]. Women with non-obstructive reproductive tract anomalies may present at different ages due to the asymptomatic nature or delayed onset of symptoms in certain conditions; an MRI is considered the gold standard for evaluating these issues to confirm the Müllerian variant, and each condition necessitates careful counseling since obstetric and gynecologic risks and consequences can vary [16]. Early detection may lower the risk of endometriosis and aid in managing associated disorders of the renal or skeletal systems, as well as complications related to subfertility and pregnancy [17].

HOX genes play a crucial role in embryonic development by regulating the patterning and differentiation of female reproductive organs, with disruptions in their expression linked to congenital uterine malformations [18]. The overlapping expression patterns of HOXA/HOXA10 and HOXA/HOXA11 are essential for uterine development and differentiation, particularly in the functional maturation of the endometrium [19]. MM lead to increased rates of infertility, first-trimester pregnancy losses, premature labor, placental retention, fetal growth retardation, and abnormal fetal presentations [20].

The genes involved in Müllerian duct and oviduct biology include transcription factors, signaling proteins, receptors, and kinases, which regulate critical processes such as mesoepithelial progenitor specification, Müllerian duct invagination, elongation, epithelial differentiation, mesenchymal multipotency, and regional differentiation into oviducts, uterine horns, cervix, and upper vagina [21]. Further research is required to explore the factors driving Müllerian placode formation, subsequent developmental stages, key molecular candidates, regulatory networks, effector pathways, and their interactions, as well as to enhance understanding through lack-of-function models and experiments for future investigations [22]. GREB1L also emerges as a promising candidate gene for the etiology of MRKH syndrome, supported by previous reports that highlight its involvement in kidney and female genital tract development; additionally, the pedigree suggests a pattern consistent with autosomal dominant inheritance with incomplete penetrance, which may be influenced by imprinting [23]. Another study with array-comparative genomic hybridization analysis on a cohort of 103 patients with Müllerian fusion anomalies identified microdeletions and microduplications in chromosomal regions 17q12, 22q11.21, 9q33.1, 3q26.11, and 7q31.1 in eight patients; the rearrangements at 17q12, which includes LHX1 and HNF1β, as well as those at 22q11.21, have been previously associated with MRKH syndrome and in other patients with Müllerian fusion anomalies, identifying new candidate genes associated with Müllerian fusion anomalies [24]. We also have to remember that exposures to endocrine disruptors during developmental periods can adversely affect developmental programming, leading to predispositions in reproductive tissues for diseases in adulthood that may also be inherited by future generations, with mechanisms believed to include epigenetic modifications [25].

The management of these pathologies should be carried out by skilled endoscopic gynecologists and involve both medical and surgical treatments tailored to each patient based on the type of MM and their fertility goals [26]. For most surgical procedures, the key measure of their value is the patient's postoperative ability to engage in healthy sexual relationships and achieve successful reproductive outcomes; in the study of Banu et al. [27], 62% of cases required surgical correction, resulting in an excellent reproductive outcome, with 26 patients (45.06%) achieving pregnancy after the intervention.

Emerging research areas, such as advancements in diagnostic techniques, innovative treatment modalities, and genetic studies, highlight the necessity for a thorough understanding of both physical and psychosocial aspects, providing valuable insights for healthcare professionals.

## Conclusions

This case of MM highlights the importance of the genetic component in the suspicion of a familial origin and its impact on improving the reproductive prognosis of affected families; MM result from various embryological mechanisms, with normal female reproductive system development relying on genetic, hormonal, and epigenetic events, any disruption of which can lead to MM; thus, a comprehensive approach that includes genetic analysis, detailed family history, and genetic counseling is recommended for identifying hereditary patterns, anticipating reproductive complications, and enhancing clinical outcomes.
